# Comparative Study of Ultrasonography and Computed Tomography in the Diagnosis of Nasal Bone Fractures

**DOI:** 10.7759/cureus.102841

**Published:** 2026-02-02

**Authors:** P. B. Anirudh, Prashanth Babu, Anil K Sakalecha

**Affiliations:** 1 Otorhinolaryngology, Sri Devaraj Urs Academy of Higher Education and Research, Kolar, IND; 2 Otorhinolaryngology & Head and Neck Surgery, Sri Devaraj Urs Academy of Higher Education and Research, Kolar, IND; 3 Radiodiagnosis, Sri Devaraj Urs Academy of Higher Education and Research, Kolar, IND

**Keywords:** clinical examination, computed tomography, diagnostic accuracy, facial trauma, nasal bone fracture, ultrasonography

## Abstract

Objective: The objective of this study was to compare the diagnostic accuracy of ultrasonography and computed tomography (CT) in the diagnosis of nasal bone fractures (NBFs) and to assess their clinical utility in the context of current evidence-based recommendations.

Methodology: This prospective analytical study was conducted in the Department of Otorhinolaryngology between January 2021 and December 2022, encompassing 126 patients with clinically diagnosed NBFs who provided informed written consent. Ultrasonographic and CT imaging were used to detect and characterize fractures. Sensitivity, specificity, positive predictive value (PPV), negative predictive value (NPV), and accuracy of ultrasonography and CT in identifying NBFs were calculated with 95% confidence intervals (CIs).

Results: Among 126 patients (mean age: 38.4 years; 90.5% male), clinical examination identified NBFs in all cases. Ultrasonography detected 121 fractures (accuracy: 96.0%), while CT detected 125 fractures (accuracy: 99.2%). Sensitivity of ultrasonography was 88.57% (95% CI: 83.2-92.5%) compared to the CT sensitivity of 97.3% (95% CI: 94.8-98.9%). Both modalities demonstrated 100% specificity. McNemar’s test demonstrated a statistically significant difference in sensitivity between CT and ultrasonography (p = 0.008). CT showed superior sensitivity in detecting undisplaced fractures (35 vs. 31 cases, p = 0.047), with no significant difference for displaced or comminuted fractures (p > 0.05).

Conclusion: While CT demonstrates superior sensitivity, particularly for undisplaced NBFs, ultrasonography offers a valuable non-ionizing alternative for initial fracture assessment in appropriate clinical contexts. Both modalities demonstrated high sensitivity for displaced fractures. However, the choice of imaging should balance the superior accuracy of CT against the safety and accessibility of USG, with awareness of operator dependency. Further prospective studies with multi-center participation and varied operator experience levels are warranted to determine optimal imaging strategies.

## Introduction

Epidemiology and clinical significance

Road traffic accidents (RTA) represent a major public health challenge in India, accounting for approximately 8% annual growth in facial trauma cases over the past decade [1]. In India, two-wheeler accidents remain the most common cause of facial injuries, contributing substantially to hospital admissions. Male patients account for the majority of nasal fracture cases, reflecting higher exposure to trauma mechanisms [2].

Nasal bone fracture (NBF) constitutes 40-50% of maxillofacial fractures and is among the most common facial injuries in the developed world [3]. The high incidence of nasal fractures among younger populations, particularly in the second and third decades of life, has significant societal and economic implications [2]. Beyond vehicular accidents, NBFs result from self-falls, athletic activities, and interpersonal violence, making this a heterogeneous injury pattern requiring careful clinical assessment. Untreated NBFs can result in aesthetic deformities and functional impairment, including nasal obstruction, breathing difficulties, and psychological distress. Treatment options range from conservative management to closed reduction (CR) or open reduction and internal fixation (ORIF), with closed reduction representing the standard of care for most cases [3].

Diagnostic approach: evolution of evidence

Contemporary evidence-based guidelines emphasize clinical examination as the primary diagnostic tool for nasal fracture assessment [4]. Physical examination findings, including midline deviation, step-off deformity, crepitus, and localized swelling, provide sufficient diagnostic information for determining the need for reduction in the majority of cases.

Role of imaging modalities

Conventional radiography has limited utility due to poor sensitivity and frequent false-negative results, particularly for subtle fractures. Imaging is indicated in specific circumstances, including complex facial trauma with concern for multi-system involvement, medico-legal documentation and litigation support, pre-reduction cosmetic assessment, or when patients are unable to undergo adequate physical examination [4].

Computed tomography (CT) remains the reference standard imaging modality for complex facial bone trauma [5]. CT provides excellent contrast resolution, multiplanar reconstruction capability, and three-dimensional reconstruction, enabling precise fracture characterization. CT is operator-independent and can evaluate associated injuries, such as orbital fractures, paranasal sinus involvement, and intracranial pathology. However, limitations include high radiation exposure, higher cost, inability to perform coronal sections in cervical spine trauma or uncooperative patients, possible artifacts in patients with dentures, and overdiagnosis of clinically insignificant fractures. Recent literature emphasizes the importance of the ALARA (As Low As Reasonably Achievable) principle, particularly in facial trauma imaging due to the proximity of radiosensitive organs, especially the crystalline lens of the eye (posing a risk for radiation-induced cataracts) and the thyroid gland, suggesting that radiation exposure should be minimized whenever non-ionizing alternatives are viable [6].

Ultrasonography (USG) has emerged as an alternative diagnostic modality with distinct advantages. These include non-ionizing radiation, which is safe for pregnancy and pediatric populations, portability allowing for bedside evaluation, real-time assessment with dynamic evaluation capability, and cost-effectiveness. It also allows for the assessment of soft tissue injuries, assistance with intraoperative reduction, and the ability to evaluate cartilaginous nasal structures [7]. Limitations of USG include operator-dependency requiring specialized training and experience, inability to assess deeper paranasal sinus involvement, challenges in signal differentiation between bone and cartilage, inter-observer variability affecting diagnostic consistency, and limited utility in complex facial trauma with multiple bone involvement [8].

Limited literature directly compares USG and CT for NBF diagnosis. Lee et al. demonstrated that high-resolution USG showed comparable accuracy to CT in a cohort of 140 patients [9]. Jank et al. reported no significant differences in orbital floor fracture detection between USG and CT [10]. However, Friedrich et al. noted that USG failed to demonstrate extension of peripheral fracture lines compared to CT [11]. Thiede et al. demonstrated superior performance of USG for lateral nasal wall fractures but found radiography superior for dorsal nasal fractures [12]. These heterogeneous findings underscore the need for prospective comparative studies in larger patient populations.

Our hospital is a tertiary care center located on the national highway NH-4, serving as the primary referral center for trauma in the district. The Department of Otorhinolaryngology manages a large volume of head and neck trauma cases with a high incidence of facial fractures. This clinical setting provides an opportunity for systematic evaluation of imaging modalities. Otolaryngologists, as specialists in nasal and airway management, are uniquely positioned to integrate imaging findings with clinical examination for optimal patient care. The specific objectives of this study were defined as follows: (i) the comparative evaluation of the diagnostic accuracy of high-resolution USG versus CT in patients with clinically diagnosed NBFs; (ii) the evaluation of diagnostic performance specifically stratified by fracture subtype (undisplaced, displaced, comminuted); and (iii) the assessment of the clinical applicability of USG as a radiation-free alternative versus CT of the paranasal sinuses (PNS) within the context of current guideline-based practice.

## Materials and methods

Study design and ethics

This was a prospective analytical study conducted in the Department of Otorhinolaryngology of Sri Devaraj Urs Medical College, Sri Devaraj Urs Academy of Higher Education and Research, Kolar, Karnataka, India, between January 2021 and December 2022. All participants provided informed written consent. The study was approved by the Sri Devaraj Urs Medical College Institutional Ethics Committee (approval number: SDUMC/KLR/IEC/651/2020-21) and was conducted in accordance with the Declaration of Helsinki principles.

Study population

The study group consisted of 126 patients who met the inclusion and exclusion criteria. Inclusion criteria comprised patients aged 10 years or older with a clinical diagnosis of NBF during the study period, confirmed by clinical examination findings, such as midline deviation, step-off deformity, crepitus, localized swelling, or deformity. Exclusion criteria encompassed patients presenting with complex fractures of the facial skeleton involving multiple bones, pregnant women, patients unable to tolerate imaging procedures, cases with incomplete imaging data, and patients under 10 years of age.

Clinical assessment and imaging protocol

All patients with suspected NBFs underwent a detailed physical examination. Clinical assessment was performed independently by an experienced otolaryngologist who recorded demographic data (age, gender, occupation), mechanism of injury, physical examination findings (swelling, crepitus, deviation, deformity), associated injuries, and nasal airway assessment.

Clinical examination by the otolaryngologist served as the primary diagnostic criterion for fracture presence or absence, reflecting current evidence-based practice. However, this approach has inherent limitations, as clinical examination cannot reliably differentiate between fracture types (undisplaced vs. displaced vs. comminuted) or assess isolated cartilage involvement. CT imaging was used as the comparative reference standard for imaging modality assessment, with acknowledgment that CT findings may overdiagnose clinically insignificant fractures.

USG Protocol

USG evaluation was performed prior to CT imaging by a senior radiologist with over 10 years of experience in musculoskeletal ultrasound. All USG examinations were performed by the same radiologist to standardize technique and minimize inter-observer variability. The equipment utilized was a PHILIPS EPIQ 5 ultrasound machine (Koninklijke Philips N.V., Amsterdam, Netherlands) with a 5-12 MHz linear transducer probe. The scanning protocol included a midline longitudinal image of the nasal bone, transverse images at three levels (upper, middle, lower), assessment of both nasal bone cortices, documentation of fracture line location, continuity, and displacement, and evaluation of surrounding soft tissue involvement.

CT Protocol

A multidetector CT scan was performed with a slice thickness of 1-2 mm. Reconstruction included multiplanar (axial, coronal, sagittal) and volume-rendered 3D reconstructions. The field of view covered the paranasal sinuses and anterior facial skeleton. CT scans were reviewed by the same senior radiologist who performed ultrasonography, ensuring consistency in interpretation methodology.

Statistical analysis

Data analysis was performed using IBM SPSS Statistics version 22 (IBM Corp., Armonk, New York, United States) [13]. Continuous variables are presented as mean ± standard deviation (SD), and categorical variables as frequencies and percentages. Diagnostic performance metrics (sensitivity, specificity, positive predictive value (PPV), negative predictive value (NPV), and accuracy) were calculated with 95% confidence intervals (CIs) using the Agresti-Coull method. McNemar’s test was utilized to compare diagnostic sensitivity and specificity between USG and CT, including subgroup analyses for specific fracture patterns. The Chi-square test was applied for categorical comparisons, with statistical significance defined as p < 0.05.

## Results

Demographic characteristics

The study included 126 patients with clinically diagnosed NBFs. Demographic and clinical characteristics are presented in Table 1. The study population was predominantly male (90.5%) with a mean age of 38.4 ± 14.2 years. The predominance of NBFs among students reflects their participation in sports and higher-risk activities. The significant representation of outdoor occupational groups (farmers, laborers) reflects occupational hazard exposure. Road traffic accidents accounted for 84.3% (n = 106) of nasal fractures, with two-wheeler accidents being the predominant mechanism (76.2%, n = 96). This finding reflects the widespread use of two-wheelers as primary transportation in India and supports the need for improved safety awareness and helmet compliance programs. Conservative management was the predominant treatment approach (73.8%), reflecting that the majority of nasal fractures (undisplaced or minimally displaced) do not require surgical intervention.

**Table 1:** Baseline characteristics of study participants (N=126)

Characteristic	Category	Frequency (Percentage)
Sex	Male	114 (90.5%)
	Female	12 (9.5%)
Occupation	Students	42 (33.3%)
	Farmers	30 (23.8%)
	Laborers	29 (23.0%)
	Clerks	14 (11.1%)
	Homemakers	9 (7.1%)
	Shopkeepers	2 (1.6%)
Mechanism of Injury	Two-wheeler rider	85 (67.5%)
	Two-wheeler pillion	11 (8.7%)
	Self-fall/slip	13 (10.3%)
	Direct facial trauma	6 (4.8%)
	Three-wheeler driver	4 (3.2%)
	Three-wheeler passenger	4 (3.2%)
	Public transport accident	2 (1.6%)
	Four-wheeler driver	1 (0.8%)
Treatment Modality	Conservative management	93 (73.8%)
	Closed reduction	27 (21.4%)
	Open reduction and internal fixation	6 (4.8%)

Imaging findings comparison

Based on a comparative assessment of USG and CT imaging, the majority of findings demonstrated concordance. However, notable differences emerged in the detection and categorization of undisplaced fractures. The comparative findings between the two modalities are detailed in Table 2. The most significant discrepancy between modalities was in the detection and categorization of undisplaced fractures. CT identified 35 cases of undisplaced fracture compared to 31 cases by ultrasonography (a discrepancy of four cases or 11.4%). USG missed these four undisplaced fractures, likely because minimal bone displacement generates minimal acoustic change. For displaced fractures (n = 84) and comminuted fractures (n = 6), both modalities demonstrated identical categorization, indicating excellent concordance for obvious fractures. 

**Table 2:** Comparative assessment of nasal bone fracture findings (N=126) USG: ultrasonography; CT: computed tomography

Fracture Categorization	USG, n (%)	CT, n (%)
Fracture not detected	5 (3.9)	1 (0.8)
Undisplaced	31 (24.6)	35 (27.7)
Displaced	84 (66.7)	84 (66.7)
Comminuted	6 (4.8)	6 (4.8)

**Figure 1:**
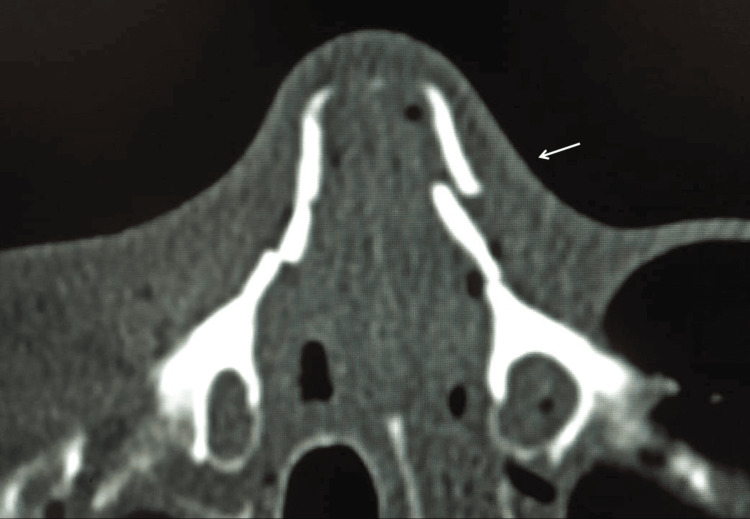
Axial computed tomography scan of a displaced nasal bone fracture The scan serves as the reference standard, clearly showing a distinct fracture of the nasal bone (arrow) with cortical disruption and displacement.

**Figure 2:**
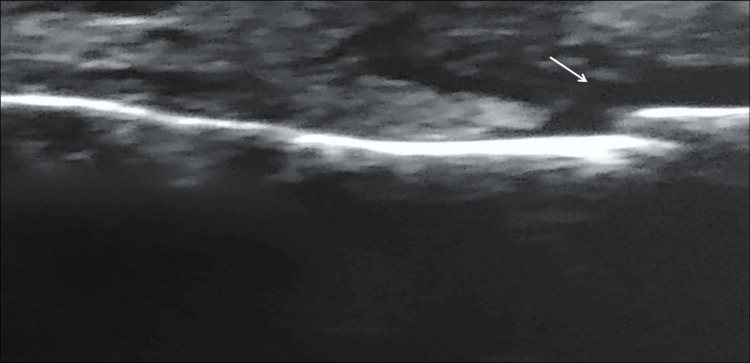
High-resolution ultrasonography of the displaced nasal bone fracture The ultrasound image corresponds to the same injury shown in the CT scan (Figure 1), demonstrating the step-off deformity and cortical break (arrow). The acoustic pattern strongly correlates with the bony displacement visualized on CT.

To visually demonstrate the diagnostic correlation between modalities, representative images of a patient with a displaced NBF are presented. Figure 1 displays the reference standard axial CT scan, revealing the distinct fracture line and bony displacement. Correspondingly, Figure 2 illustrates the high-resolution USG findings, highlighting the capability of this modality to visualize the cortical disruption and step-off deformity in correlation with the CT findings.

Diagnostic accuracy

CT

CT demonstrated superior sensitivity for detecting NBFs in this cohort. The diagnostic performance metrics for CT are presented in Table 3. The single missed fracture by CT (n=1) occurred in a patient with minimal bone displacement on clinical examination. Specificity was 100%, and the high PPV (100%) indicates that all fractures detected by CT were confirmed clinically.

**Table 3:** Diagnostic performance of computed tomography

Diagnostic Measure	Value	95% Confidence Interval
Sensitivity	97.30%	94.8 - 98.9%
Specificity	100%	81.5 - 100%
Positive Predictive Value	100%	98.0 - 100%
Negative Predictive Value	50.00%	15.7 - 84.3%
Accuracy	99.20%	96.0 - 99.9%

USG

USG demonstrated 88.57% sensitivity, missing five of 126 clinically diagnosed nasal fractures. The diagnostic performance metrics for ultrasonography are presented in Table 4. All fractures detected by USG were confirmed clinically (PPV = 100%), indicating no false-positive results.

**Table 4:** Diagnostic performance of ultrasonography

Diagnostic Measure	Value	95% Confidence Interval
Sensitivity	88.57%	83.2 - 92.5%
Specificity	100%	81.5 - 100%
Positive Predictive Value	100%	97.2 - 100%
Negative Predictive Value	0%	0.0 - 52.2%
Accuracy	88.89%	82.4 - 93.6%

Statistical Comparison Between Modalities

McNemar’s test demonstrated that CT had statistically significantly higher sensitivity than ultrasonography for detecting NBFs overall (p = 0.008). The absolute difference in sensitivity was 8.73 percentage points.

Subgroup analysis by fracture type

CT showed statistically superior sensitivity for undisplaced fractures (100% vs 88.6%; p = 0.047). USG missed 4 undisplaced fractures, likely due to minimal acoustic differentiation from normal cortex. Both modalities demonstrated equal performance for displaced fractures and comminuted fractures, with a 100% detection rate (p = 0.99). The subgroup analysis of sensitivity by fracture pattern is summarized in Table 5.

**Table 5:** Subgroup analysis of sensitivity by fracture pattern

Fracture Pattern	Modality	Detected / Total	Sensitivity (95% CI)	Statistical Test	Test Statistic (χ²)	p-value
Undisplaced	USG	31 / 35	88.6% (72.6 - 96.2%)	McNemar's Test	4.00	0.047
	CT	35 / 35	100% (91.2 - 100%)			
Displaced	USG	84 / 84	100% (95.4 - 100%)	McNemar's Test	0.00	0.99
	CT	84 / 84	100% (95.4 - 100%)			
Comminuted	USG	6 / 6	100% (54.1 - 100%)	McNemar's Test	0.00	0.99
	CT	6 / 6	100% (54.1 - 100%)			

## Discussion

Comparison with established literature

Our findings align with established literature while offering critical refinements regarding fracture subtypes. Lee et al. (2009) reported a high-resolution USG sensitivity of 94.3% in a cohort of 140 patients [9]. Our observed sensitivity of 88.57%, while comparable, is slightly lower. This marginal discrepancy may reflect variations in patient population, specifically the prevalence of subtle fractures in our cohort or the inherent operator-dependency of ultrasonography.

Furthermore, our study clarifies conflicting reports regarding the equivalence of USG and CT. While Jank et al. (2004) [10] and Kishibe et al. (2005) [14] concluded that the two modalities were largely indistinguishable, our subgroup analysis provides necessary nuance to these broader claims. We demonstrated that while the modalities are indeed indistinguishable for "obvious" injuries (displaced and comminuted fractures), significant performance gaps emerge specifically in undisplaced fractures. This suggests that the "equivalence" noted in earlier studies likely depends heavily on the severity of the injury patterns included in their cohorts. Additionally, while Thiede et al. (2005) [12] highlighted the superiority of USG specifically for lateral nasal wall fractures, our data indicate that USG maintains a consistent sensitivity profile across general nasal trauma, validating its utility as a primary screening tool.

Contextualization with recent evidence (2024-2025)

When positioned against recent systematic reviews, our results highlight the impact of patient demographics and setting on diagnostic accuracy. A 2024 meta-analysis of pediatric nasal fractures (four studies, n=277) reported a lower USG sensitivity range of 66-78% [15]. Our adult cohort achieved significantly higher sensitivity (88.57%), suggesting that the larger anatomical structures in adults, combined with high-frequency transducers, allow for greater diagnostic clarity than is possible in pediatric populations.

Similarly, recent investigations into adult fractures in community settings (2024) have reported sensitivities of 71-73% for both modalities [16]. The superior performance observed in our study likely reflects the tertiary care environment, where examinations were performed by experienced radiologists rather than general practitioners. This underscores that while USG is a powerful tool, its diagnostic ceiling is closely tied to operator expertise and the clinical setting.

Fracture pattern analysis

Subgroup analysis demonstrated that CT is superior for undisplaced fractures (100% vs. 88.6%, p=0.047), as USG struggles with minimal acoustic differentiation. However, this statistically significant difference has limited clinical impact, as undisplaced fractures are managed conservatively and were identifiable via physical exam (swelling/crepitus). Importantly, both modalities achieved 100% accuracy for displaced and comminuted fractures, the injuries requiring intervention.

Modality comparison

USG offers distinct advantages, including a lack of ionizing radiation, cost-effectiveness, portability, and real-time dynamic capability (useful for reduction). However, it remains operator-dependent and limited by a narrow field of view. A specific practical challenge noted with USG is the difficulty related to local tenderness; manipulating the transducer over an acutely fractured, edematous nasal dorsum can elicit significant pain. This discomfort may limit the operator's ability to apply necessary pressure for optimal acoustic coupling or cause motion artifacts, a limitation not encountered with non-contact CT imaging. To mitigate these risks in our study, a "stand-off" technique was employed using a copious amount of acoustic coupling gel, allowing the radiologist to obtain images with minimal direct pressure on the compromised bony framework. Conversely, CT serves as the sensitive, operator-independent reference standard ideal for surgical planning and complex trauma, though it is constrained by radiation exposure, cost, and the potential for overdiagnosing clinically insignificant findings. Critically, imaging results rarely altered clinical management, which was dictated by physical assessment in the vast majority of cases (73.8% conservative; 21.4% closed reduction). Imaging served primarily as a confirmatory tool, becoming essential only for the 4.8% of patients requiring ORIF for complex trauma. This supports current guidelines emphasizing that radiographic assessment should not delay clinical decision-making.

Limitations

A critical limitation of this study is the reliance on a single senior radiologist (>10 years of experience). While this minimized inter-observer variability, it restricts generalizability to non-specialized settings. Consistent with literature highlighting the significant impact of operator experience [10] and inter-examiner inconsistency [11] on USG performance, our high sensitivity metrics may not be reproducible by less experienced operators. Future multi-operator trials are essential to establish valid accuracy profiles for diverse clinical environments. Additionally, a critical limitation is the ambiguity of the reference standard; utilizing clinical examination as the inclusion criterion assumes diagnostic infallibility, potentially introducing bias where soft tissue edema mimics osseous injury despite negative CT findings. Furthermore, despite highlighting the theoretical advantages of ultrasonography, this study did not formally quantify procedure time, patient pain scores (VAS), or cost-benefit ratios to validate practical utility beyond diagnostic accuracy. Finally, as this was a cross-sectional study focused on initial diagnostic accuracy, longitudinal follow-up with repeat investigations to document the radiological process of fracture healing was not performed. While ultrasonography offers a potential radiation-free modality for monitoring healing, this was outside the scope of the current analysis.

Current clinical recommendations and role of imaging

Current guidelines from the American Academy of Otolaryngology-Head and Neck Surgery [17], the Canadian Medical Association [4], and ENT UK [18] posit that physical findings such as midline deviation, crepitus, and step deformity are often sufficient for diagnosis, reserving imaging for complex or equivocal cases. Within this established framework, our study focused on optimizing the selection of imaging modalities when radiographic assessment is deemed necessary. Our findings suggest that ultrasonography fits well into these low-radiation protocols, offering a safe alternative to CT for fracture characterization without disrupting the emphasis on time-sensitive interventions like closed reduction. 

## Conclusions

This prospective comparative study confirms that while CT achieves superior sensitivity (97.3%, 95% CI: 94.8-98.9%) compared to USG (88.57%, 95% CI: 83.2-92.5%), particularly for undisplaced fractures, both modalities demonstrate equivalent performance (100% sensitivity) for displaced and comminuted fractures. Consequently, for patients presenting with clinical signs of nasal trauma, USG functions as a viable, radiation-free alternative for characterizing fracture patterns and determining the need for reduction, whereas CT should be reserved for complex cases or when ultrasonography findings are equivocal.

USG offers a valuable, non-ionizing alternative for initial fracture screening, especially in pediatric and pregnant populations, though its utility is constrained by operator dependency and lower sensitivity for subtle, undisplaced fractures. Additionally, as this data reflects a single-center experience with an expert radiologist, the reproducibility of these high accuracy rates in non-specialized settings may vary. In contrast, CT remains the reference standard for complex facial trauma and medico-legal documentation but carries risks associated with radiation exposure and higher costs. Ultimately, imaging should be employed to support, not supersede, clinical judgment, ensuring that acute management decisions are not unnecessarily delayed.
